# Supplementary material for: Are carbon emissions trading and green financial instruments synergistic? -Comprehensive quantitative research based on content analysis
Source: PLoS One. 2024 Mar 7;19(3):e0298601. doi: 10.1371/journal.pone.0298601 (PMC10919601; doi:10.1371/journal.pone.0298601)
Supplement: S1 Data — (ZIP) [file pone.0298601.s001.zip › Data/地级市Z_F工作报告.zip!/╜¡╦╒/╒≥╜¡╩╨/└┤╫╘╣1⁄2╓┌║┼í╛┬φ┐╦╩2╛▌═°í┐_2015.docx]

各位代表：

现在，我代表镇江市人民政府向大会作工作报告，请予审议，并请各位政协委员提出意见。

2014年工作回顾

刚刚过去的一年，是极不平凡的一年。一年来，在省委、省政府和市委的正确领导下，我们紧紧依靠全市人民，全面贯彻落实党的十八大和十八届三中、四中全会以及习近平总书记系列重要讲话精神，坚持稳中奋进、着力改革创新，经济社会发展取得了新成绩，较好完成了市七届人大三次会议确定的各项任务。

回顾过去的一年，五个鲜明特点令人印象深刻：

——这是镇江在国际国内产生较大影响的一年。习近平总书记12月13日亲临镇江视察，镇江的山山水水、历史文化和创业创新精神，给总书记留下很深印象，称赞“镇江很有前途”；低碳城市建设做法被国家推荐到联合国作介绍；《福布斯》中国大陆城市创新力排行榜镇江列第19位；赵亚夫同志被评为全国时代楷模。镇江的知名度和影响力大幅提升，全市人民的自信心和自豪感显著增强。

——这是在应对各种挑战中奋力作为的一年。积极面对经济下行、产业转型、企业运行中的各类压力和困难，坚持稳中奋进、多措并举，全年预计实现地区生产总值3220亿元，增长10.5%；公共财政预算收入277.8亿元，增长9.1%；税占比82.4%，居全省第五；固定资产投资2142.3亿元，增长22.2%；城乡居民两个收入分别达35695元和17622元，增长9%和11%；全国文明城市创建获得省级测评第一。

——这是重大项目和载体建设快速推进的一年。北汽项目全面开工，惠龙e通(1)上线运行，连淮扬镇铁路镇江最优方案成功获批，华东地区首个通用机场——大路通用机场正式开飞；跻身苏南国家自主创新示范区，创成国家级高新技术产业开发区，中瑞生态产业园正式开园，产业“三集”(2)发展扎实推进。

——这是生态文明建设成效显著的一年。成为国家生态文明建设先行示范区和全省唯一的生态文明建设综合改革试点市，低碳城市建设管理云平台在全国乃至全球低碳城市建设中产生广泛影响，“两个片区”(3)、“一湖九河”(4)整治取得较好成效。

——这是重点改革任务有力落实的一年。在全省率先实施主体功能区制度、率先进行市级投融资平台改革、率先开展项目“多评合一”(5)。建立健全规划审查、产业项目评估、长江岸线资源管理、信息化投资和政府投资项目全过程管理等制度。

通过一年的不懈奋斗，取得了八个方面成效：

一是经济保持平稳增长。213个市级重点产业项目完成投资700亿元；全市技改投入353亿元、增长33.9%。开展“三集”、文创产业等6场专题招商，产业类实到外资12.2亿美元，吸引民资447亿元。开展“帮困解难服务季”活动，召开12次企业家圆桌会议，举办3场“企业家沙龙”，共为248家企业解决300多个问题；积极促进小微企业发展，共减税3.7亿元；规模以上工业企业实现利税772亿元、利润503亿元，分别增长14.9%和16.2%。新增上市企业1家、挂牌企业10家，成功开展全省第一笔小额贷款资产证券化业务，全市社会融资规模突破900亿元 落霞小说。充分发挥政府性债务管理领导小组作用，建立政府偿债准备金和政府投资项目两个“资金池”，严控债务增长；立足防范金融风险，一些企业因互保产生的出险问题得到有效化解。

二是产业加快转型升级。产业“三集”园区完成投资1100亿元，新开工重大产业项目41个、在建56个，销售收入增长15%，首批6个试点园区启动市场化运作。高端装备制造、新材料等新兴产业销售收入增长25%以上。服务业投资增长36.6%，增幅居全省第一；服务业增加值占GDP比重达45%，同比提高1.2个百分点。旅游总收入和物流业、文化产业营业收入分别增长16%、20.1%和15%，句容成功承办全省乡村旅游节，茅山风景区创成国家5A级景区。新增高效设施农业面积5.9万亩，粮食生产实现“十一连增”，四个小型农田水利重点县项目绩效考评全省领先。高新技术产业产值占规模工业产值比重达48%，保持全省第一；研发经费支出占GDP比重达2.55%；新增国家“千人计划”28人、省“双创计划”44人，市“331计划”第七批确定资助160人；引进本科以上人才13980人。

三是生态建设扎实推进。创成国家生态市、国家森林城市，荣获中国人居环境奖。低碳城市“九大行动”(6)126个项目顺利推进，对48家重点企业实施碳资产管理，对166个固定资产项目实施碳评估，单位GDP二氧化碳排放强度同比下降4.8%。开展“两个片区”环境综合整治，东部谏壁片区治理污染企业28家、关停搬迁6家，群众搬迁工作开始启动，镇澄路西段拓宽改造工程竣工通车；西南韦岗片区关闭企业141家，凤凰山山体公园整治和十里长山采石宕口治理工程基本完成，243省道沿线整治完成年度任务。“一湖九河”水环境综合整治扎实推进，铺设截流管网42公里，完成河道清淤180万立方米，引水换水近1亿立方米，新建河滨绿化面积14万平方米，基本实现市区水系互连互通。开展长江镇江水域综合整治。实施 117项大气污染防治工程，市区空气质量良好以上天数占比达65.9%，PM2.5(7)平均浓度下降6.25%。

四是城乡面貌不断改变。召开了17次规划审查会、25次专题审查会，对262个项目进行审查把关。完成城市总体规划修编纲要。市区实施城建项目218个，完成投资580亿元，镇江至扬中快速通道、南山北入口景区、大西路东段拓宽改造等30项重点竣工项目全面完成，“7+1”旧城区城中村改造(8)全力攻坚，五凤口高架有序推进。圆满完成市区人力客运三轮车退市工作，渣土车和犬类整治取得阶段性成效。市区拆除违建53.7万平方米，新投入公共自行车3000辆。丹阳主城成片开发改造成效显著，完成人民大桥重建、万善公园扩建。句容沪宁高速城区互通建成通车，客运交通枢纽投入运营。扬中建成奥体中心和三桥，“四城同创”(9)取得突破。全市65个三星级康居乡村通过省级验收，建成1个“美丽宜居镇”、10个“美丽宜居村庄”。

五是改革红利逐步释放。坚持市场化改革方向，7家市级投融资平台重组为“一中心、四集团”(10)。公布市级行政权力清单和行政审批事项、行政事业性收费目录清单，行政审批事项压缩74%、审批周期压缩2/3以上。在全国口岸率先取消代理报检收费。实施商事登记制度改革，新设立民营企业8893户、注册资本404亿元，分别增长14.5%和26.2%。丹阳省级农村改革试验区和中农办农村改革实验联系点、扬中农村土地承包经营权确权登记颁证试点扎实开展。医药卫生体制改革、教育综合改革进一步深化。新一轮文化体制改革全面启动。

六是群众生活持续改善。全市可用财力的71.2%、新增财力的81.5%用于保障和改善民生。“百村万户”双达标行动(11)扎实开展，80%的经济薄弱村和低收入农民达标。新增城镇就业7.98万人，扶持创业1万余人，城镇登记失业率为1.91%。城乡基本保障覆盖率达97.6%，低保标准提高到每人每月580元。开工各类保障性住房19161套、建成11610套。完成校安工程85.9万平方米，校车公交化实现全覆盖，辖市区全部通过国家义务教育基本均衡县评估验收。全市二级以上医疗机构均设立健康管理与促进中心，康复联合病房全面推广。创成省级公共文化服务体系示范区，文化广场标准化建设入选全国文化创新工程。成功承办2项国际级、3项国家级体育赛事。新建改造7个菜市场，全面完成100家平价商店建设。

七是社会建设成效显著。城乡和谐社区达标率均达93%。全力组织和开展“五大专项行动”(12)，顺利完成南京青奥会、国家公祭日等重要活动保障任务，公众安全感列全省第1位，信访积案化解走在全省前列。审计工作全力服务大局，重大项目和民生工程审计进一步加强。食品药品信息化监管中心正式运行，“舌尖上的安全”共治经验在全国推广。圆满完成第三次全国经济普查。省级“双拥”模范城创建实现辖市区“满堂红”，成功创建省“诗词之市”，再次获得全国慈善七星级城市称号。外事、侨务、人口计生、民族宗教、机关事务等工作取得新成绩，编制、档案、地方志、防震、气象、供销、科普、老龄、妇女儿童、关心下一代、残疾人、红十字等事业取得新进展，国家安全、国防教育、人民武装、人民防空、援藏援疆等工作取得新成效。

八是自身建设切实加强。以“一满意、三到位、见实效”(13)为目标，市政府党组党的群众路线教育实践活动扎实开展，对查摆出的问题逐一整改落实，并完善10项制度；组织开展市级机关“一把手”国防教育和纪律训练，政府系统作风建设取得实效。不断加强民主法制建设，坚决贯彻市委“三重一大” (14)事项决策制度，自觉接受市人大及其常委会的监督，坚持重大事项向市人大常委会报告、向市政协通报，充分发挥市政协和各民主党派、工商联、无党派人士以及人民团体的民主监督、协商议政作用。政府系统承办人大代表建议313件、办理政协提案448件，满意和基本满意率达100%。深化法治政府建设，完善规范性文件和重大决策合法性审查机制，健全政务公开和定期新闻发布制度，推进行政权力网上公开透明运行，被评为全省“六五”普法中期先进城市。严格执行中央“八项规定”和省委、市委“十项规定”，全面完成年初确定的政府系统廉政建设16项任务，“三公”经费(15)同比下降36%，下发文件同比减少33%。

各位代表!回顾过去一年，成绩令人鼓舞!这些成绩，归根结底是全市上下奋发进取、齐心协力干出来的!在此，我们要为全市人民点赞，为广大基层干部叫好，向各位人大代表、政协委员，各民主党派、工商联、无党派人士、人民团体，驻镇人民解放军、武警官兵、人民警察，离退休老同志和所有关心支持镇江发展的海内外朋友们致敬!

与此同时，我们也清醒地认识到，工作中仍然存在一些问题，主要是：重大项目支撑乏力，一些企业生产经营困难，产业转型升级任务依然很重；创新能力不强、创业氛围不浓、开放程度不高；中心城市的辐射带动能力不强，破解新型城镇化瓶颈制约的有效办法不多；生态环境与群众的期望值仍有差距；机关工作中“慵懒散”“软浮拖”现象仍然存在，公务人员的职业水准有待提高。对以上这些问题，我们将高度重视，扎实加以解决。2015年主要任务

今年是全面深化改革的关键之年，是全面推进依法治国的开局之年，也是全面完成“十二五”规划的收官之年。按照省委十二届八次、九次全会和市委六届九次全会的部署，今年政府工作的总体要求是：全面贯彻党的十八大和十八届三中、四中全会、中央经济工作会议以及习近平总书记视察镇江时的重要讲话精神，围绕“五个迈上新台阶”(16)，坚持生态领先、推进特色发展，以提高发展质量和效益为中心，主动适应引领经济发展新常态，狠抓改革攻坚，突出创新驱动，强化风险防控，加强民生保障，自觉依法行政，加快建设经济强、百姓富、环境美、社会文明程度高的新镇江。

做好今年工作，要做到“四个牢牢把握”：

——牢牢把握习总书记对江苏发展的新要求。切实把思想和行动统一到总书记的重要讲话精神上来，以“迈上新台阶、建设新江苏”为目标，全面提高镇江小康建设的质量和水平，为基本现代化建设奠定更加坚实的基础，使发展成果经得起实践、人民和历史的检验。

——牢牢把握经济发展新常态的趋势性变化。深刻理解新常态带来的九个趋势性变化，切实以创新发展为重要抓手，以转方式、调结构为鲜明导向，积极鼓励并大力发展新技术、新业态、新模式，主动适应产业组织方式小型化、智能化和专业化的变化，营造充分竞争的市场环境，在创新中增创新优势、培育新增长点、提升发展质量，真正使创新成为驱动镇江发展的新引擎。

——牢牢把握“生态领先、特色发展”的战略定位。认真贯彻市委六届九次全会精神，在全面落实主体功能区制度、打造全国第一朵“生态云”(17)、实施重大片区环境整治、率先关闭“五小”企业(18)、推进生态建设等方面领先；在产业“三集”、发展“海陆空”(19)高端装备制造、新材料和现代旅游等产业、建设山水花园城市、深化医药卫生体制改革、实施特困群体托底保障工程、推行“多评合一”、试点“多规合一”(20)等方面，形成鲜明特色，做到人无我有、人有我优、人优我特。

——牢牢把握稳中奋进的总基调。积极应对新挑战、增添新动力、推动新发展，确保优势指标保持全省第一方阵，主要指标高于全省平均水平，特色指标力争走在前列。全年地区生产总值、一般公共预算收入增长9%左右，税占比进一步提高；固定资产投资增长16%左右；社会消费品零售总额增长13%；全面完成省下达的节能减排约束性指标。

工作中，将着力抓好六个方面：

一、推进生态文明建设走在前当示范

优化空间布局。加强制度设计，强化系统推进，加快建设国家生态文明先行示范区，深入推进省生态文明建设综合改革试点，认真抓好主体功能区产业准入、环境准入和分类考核等6个配套文件的落实，坚决执行最严格的耕地保护和节约集约用地制度，把该保护的保护好、该发展的发展好，加快构建“一区、一网、三带、十载体、多节点”(21)的生态布局，努力形成高效、集约、均衡、永续的发展格局。

推进低碳发展。实施新一轮低碳“九大行动”120项任务，加快低碳产业、低碳园区、低碳景区、低碳校园、低碳机关建设，大力推进“金屋顶”计划(22)，新建筑节能标准执行率达100%，力争率先创成全国低碳模范城市。构建循环经济体系，50%以上的省级园区完成循环化改造任务；对照省定标准，对市级园区开展循环化改造。深入开展化工企业专项整治，对其中无法改造升级的20家予以关闭。

彰显青山绿水。对全市235座山体全面加强保护，推进矿山企业关闭和宕口复绿；对主城区尚未改造的14座山体分批实施改造修复。完成“一湖九河”水环境综合整治，实施苏南运河谏壁段生态修复工程；实行严格的水资源管理制度，全市地表水断面水质好于III类比例达70%以上。推进林业“五项工程”(23)，新增造林面积1.5万亩，建成省级绿化示范村55个；续建润扬大桥生态园一期，实施京江绿道、南山绿道配套工程，新建一批街头绿地，城市绿化覆盖率达42.5%。加快推进焦东滩生态湿地、赤山湖国家湿地公园建设，全市自然湿地保护率达43%以上。

加强环境治理。东部谏壁片区加快金港产业园区建设，积极开展项目招引；重点推进25家企业治污，完成天龙化工搬迁，启动雩山水泥厂关停、丹徒化肥厂搬迁；稳步推进谏壁集镇建设、居民搬迁等工作。西南片区关闭128家小土窑、小水泥、小轧石厂等企业；继续抓好道路扬尘管控，对243省道等超载超限问题加大治理力度。强化大气污染防控，狠抓粉尘、尾气、扬尘和油烟等四类专项整治，扎实做好秸秆综合利用和禁烧、工业污染防治等工作。

二、着力构建现代产业体系

加快新型工业化步伐。重点实施3亿元以上先进制造业项目87个，当年完成投资390亿元。壮大优势产业。高端装备制造产业加快推进中船船舶与海工配套产业园、中陆航星产业化生产基地等34个项目，北汽华东镇江基地第一辆车下线。新材料产业突出高性能合金、碳纤维、化工新材料，加快推进恒神碳纤维及其复合材料等16个项目，产业规模突破1100亿元。培育新兴产业。围绕医疗影像诊断设备、新能源汽车、工业机器人和智能制造等，大力推进鱼跃医疗设备、生物工程设备与生物制药等55个项目，集聚关联企业，完善产业链条。改造传统产业。运用清洁生产技术、信息化技术和节能降耗工艺，改造提升轻工、建材、造纸等传统产业，淘汰35家企业落后产能，培育20家省级以上“两化融合”试点示范企业，工业技改投资突破400亿元。

提高现代服务业发展水平。重点实施5亿元以上现代服务业项目88个，当年完成投资350亿元以上。现代旅游业实现综合收入620亿元，启动西津渡5A级景区创建，加快丹徒世业洲、丹阳水晶山、句容茅山湖三个省级旅游度假区和扬中雷公岛的规划建设，加大温泉等旅游资源开发，建成30个省级以上休闲农业示范点。现代物流业完成增加值238亿元，重点推进润祥石油产品物流园等18个项目；支持惠龙e通做大做强网上生产资料市场，力争营业收入突破700亿元。文化产业实现营业收入560亿元，重点推进台江软件园、国家数字出版基地镇江园区等40个项目。同时，大力发展楼宇经济、总部经济、平台经济等新型业态，加快培育金融、电子商务、云计算、物联网、健康养老等新兴产业，提升发展层次。

推动现代农业高效发展。重点实施5000万元以上现代农业项目15个，当年完成投资15亿元。深化农业结构调整，突出优质粮油、特色园艺、特种养殖、高效林业、休闲农业五大产业，大力发展生态农业，推广绿色食品、有机食品生产技术。积极培育农业新型经营主体，新增省、市两级示范家庭农场50家、“五好”示范社(24)50家，新增省级以上农业龙头企业5家，全市新型农业经营主体规模经营比重达47%。深入推进句容国家现代农业示范区建设，镇江新区“中国——意大利农业创新示范园” 初步展示形象。“亚夫在线——镇江农村合作经济联盟”上线运营。

全力打造特色载体。狠抓“三集”园区的项目招引、市场化运作和功能配套，抓成15个总投资30亿元以上的先进制造业项目和20个总投资20亿元以上的现代服务业项目，产业集中度达60%以上。广泛开展国际合作，按照“三基地、三中心”(25)的定位，纵深推进中瑞生态产业园建设；着力推进生态文明先行区试点园区建设；海峡两岸新材料产业合作示范区等载体引进一批新项目；创成国家级综保区。加快港产城融合发展，在全市域范围优化配置岸线、货物等资源，编制镇江新区、扬中、丹阳沿江“金三角”区域一体化发展规划，加快推进相关项目。支持丹阳创建国家级开发区。

全面扩大对外开放。主动对接“一带一路”(26)战略和长江经济带建设，推进江海河联动，大力开展以商引商、中介招商、代理招商和小分队招商，力争产业类实际利用外资13亿美元。引导外资进入医疗卫生、健康养生、生态环保和教育文化等领域，鼓励引进风投、创投等金融资本。加快推进“三个一”联合通关(27)，加强海事监管现代化镇江示范区建设，支持企业在上海自贸区设立实体。鼓励企业“走出去”建设境外产业园、开展跨国并购、设立研发机构。

三、精心打造山水花园城市

完善城市功能。突出“精、准、成”，完成城建投资620亿元，其中市区395亿元、实施项目127项，着力彰显山水花园城市特色。全面完成城市总体规划修编。启动和平路西等棚户区征收拆迁；改造街巷1.5万平方米、积水区10处。完成大港水厂一期土建、征润洲和谏壁污水处理厂改扩建主体工程，增强城市吸水、蓄水、净水和释水功能，争创国家“海绵城市”建设试点；实施官塘变、解放变等输变电工程；推进智慧城市和大数据中心建设，完成宽带提速和移动通信网络扩容升级。扎实推进重点板块开发，南徐片区加快商务 A区招商和商务C区建设；官塘片区完成路网工程；高校园区建成共享区体育馆和图书馆，市委党校、省交通技师学院、镇江高专新校区全部竣工，江苏科技大学新校区、江苏大学京江学院局部完工。

统筹城乡发展。推进就地城镇化，深化户籍制度改革，推动市区和各辖市中心城区做大做强，增强对人口和资源的集聚力，力争常住人口城镇化率达67%。丹阳加快八佰伴等城市综合体建设，促进产城融合发展；句容开发建设“四个跨界新市镇”(28)，争当宁镇扬同城化发展先导区；扬中创成国家卫生城市、国家园林城市 侯卫东官场笔记。对规模较大、经济较发达的重点镇试行扩权升格，对一般建制镇引导因地制宜发展，打造农民转市民的主平台。构建“大交通”格局，五凤口高架力争竣工，312国道城区改线段主线贯通，建成沪宁高速丹阳东互通。连淮扬镇铁路上半年全线开工，镇江长江大桥启动建设，镇江枢纽站和新区站做好各项前期工作。完善大路通用机场集疏运体系。打造美丽宜居镇村，推动燃气、电力、污水处理等公共设施向农村延伸，加快推进城乡教育、医疗、社会保障等公共服务均等化。突出传统文化和民俗风情，推进宝堰古镇整体开发，加强对九里、儒里、华山等传统村落的保护。建成1个“美丽宜居镇”、10个“美丽宜居村庄”。

提升管理水平。调整城市管理行政执法体制，在各辖区设立行政执法局，实行属地管理、两级主体、分级负责。主城区8个街道范围全部实行清扫保洁市场化。深入开展“931”城市环境综合整治(29)，集中整治占道经营、违法建设等顽症，扎实解决“停车难”问题，进一步提升小区物业管理水平。

四、大力实施创新驱动战略

狠抓国家高新区建设。抢抓苏南国家自主创新示范区建设重大机遇，一年打基础、二年出形象、三年上台阶，着力建设创新型特色园区。实行市场化运作，建设科技创新一条街，建成船舶海洋工程装备研究院、清洁水技术应用研究院。积极培育生态环保产业，加快发展船舶与海工配套等主导产业，力争科技金融中心和知识产权交易市场、技术交易市场挂牌运营。充分放大政策优势，探索“一区多园”模式，辐射带动全市经济转型升级。

推动企业自主创新。深入推进创新型试点城市建设，实施56个科技创新重点项目，完成年度投资15.8亿元。深化企业与高校院所合作，提升知识产权创造、运用、保护和管理水平，新增省级以上研发机构40家、省产学研协同创新基地1-2个。引导企业开展科技攻关，积极承担国家、省重大科技专项，力争新认定高新技术企业60家、省级高新技术产品200项，高新技术产业产值占比保持全省领先。

壮大科技人才队伍。实施高端人才集聚、基础人才培养、优秀人才扶持、人力资源管理提升“四大计划”，着力打造区域人才高地。全年培育引进创业创新领军人才100名、企业经营管理人才1000名、高技能人才6000名。高度重视科技型企业家、民营企业传承人以及技术经纪人等队伍建设。

五、全面深化重点领域改革

深化行政管理体制改革。完成市级及辖市区政府机构改革，整合部门内设机构，提升服务效能。深化事业单位人事制度改革，推进公务员分类管理。完成“加强事中事后监管”省级试点任务，支持镇江新区推进行政审批制度改革省级试点。

深化生态文明建设综合改革。全面落实《关于推进生态文明建设综合改革的实施意见》，着力在建立健全主体功能区制度和资源有偿使用制度、实施严格的环境准入和生态保护、推进绿色低碳发展、创新管理体制机制等方面积极探索，形成可复制、可推广的“镇江经验”。

深化市场化改革。加快金融创新步伐，拓宽融资渠道，大力推广PPP模式(30)，在城建、民生、生态建设、社会事业等领域吸引社会资本参与。深化公立医院改革，推动两大医疗集团实体化运作。深入推进教育综合改革和国家学前教育体制改革，构建“管办评”分离模式。完成市属平台公司资产审计、划拨等工作，加快股权多元化改造，健全科学考核机制，推动建立现代企业制度。深化资源、环境价格等改革；健全政府购买公共服务制度。

深化农村综合改革。全面推进农村土地承包经营权确权、农村宅基地确权、农房确权、农村集体经济确权工作，推动农村集体经营性建设用地六、着力加强民生和社会建设

增加居民收入。多渠道增加城乡居民工资性、经营性、财产性和转移性收入，保持城镇、农村居民收入增长与经济增长同步。认真做好大学生、失业人员就业工作，扶持创业7000人，城镇登记失业率控制在3%以内。推进机关事业单位养老金并轨，全市社会保险主要险种参保率稳定在98%以上。积极探索“医养结合”的养老服务体系，全面完成农村敬老院改造，全市社会投资兴办养老机构床位占比达50%以上。

完善公共服务。进一步弘扬尊师重教的社会风尚，积极推进教育优质均衡发展，取消市属公办普通高中择校生，整合市区中等职业教育资源，加快江苏航空职业技术学院筹建步伐。构建分级诊疗制度，切实加强基层医疗卫生机构和乡村医生队伍建设，社区卫生机构门急诊占比达55%以上，乡镇卫生院住院占比达35%以上；加快建设“放心大药房”。完成食品药品实验室升级改造。扎实推进“书香镇江”建设，举办“长江音乐节”、“文心阅读节”等文化惠民活动。全力创建省级公共体育服务体系示范区，启动市体育运动学校改扩建工程，推进学校体育场地向社会开放，人均拥有公共体育设施面积2.7平方米以上。

创新社会管理。以文明城市建设为抓手，更好发挥志愿者作用，打造“大爱镇江”等特色品牌，大力发展慈善事业。巩固提升“网格+网络”社区管理服务体系，新建4个社区服务中心，城乡和谐社区达标率均达96%以上。深化全民国防教育，推进军民融合深度发展，确保双拥“七创”圆满成功。完善社会诚信体系，建成个人信用库，企业法人信用信息实现对社会公开查询，建立和完善失信人员联合惩戒机制。深入推进“平安镇江”“法治镇江”建设，全面完成“六五”普法任务，依法及时化解各类信访矛盾，加强公安基层所队建设，进一步营造和谐稳定的社会环境。认真贯彻新《安全生产法》，严格落实安全生产责任制，切实增强应对和处置突发事件的能力。

今年，将集中力量重点办好10件可观可感的民生实事：

1.市、辖市区、镇(街道)、村(社区)四级联动，实施特困群体托底保障工程。

2.在主城区新建4个老年日间照料中心、40个老人助餐点和10个生活无着流浪乞讨人员救助点，对“三无”老人提供免费午餐。

3.市区改造危旧房和空斗墙8万平方米，完成门牌整治任务。

4.建设征润洲水源地原水水质安全保障工程，加强金山湖应急水源地保护，确保市民饮用优质、干净、放心水。

5.完成校安工程69万平方米。

6.省级示范乡镇卫生院比例达50%以上，基层医疗卫生机构“配药难”等问题得到较好解决。

7.新建改建农村公路280公里；整治城市河道29公里、乡村河道186公里。

8.一村(社区)一法律顾问全覆盖；建设66个基层综合文化服务中心。

9.PM2.5平均浓度比上年下降3%，空气质量良好以上天数占比达66%。

10.全面完成“百村万户”双达标任务，所有低收入农民年收入达6000元。

各位代表，今年还有一项重要工作，就是研究编制“十三五”规划。我们将围绕“生态领先、特色发展”，突出前瞻性、导向性和可行性，编制一个适应时代要求、体现中央和省里精神、符合市情民意的高质量规划。

加快建设服务型政府

面对经济发展和作风建设的新常态，我们将认真落实全面从严治党的新要求，加快提升执行力、公信力和服务能力，扎实推进政府治理能力现代化。

我们一定牢记宗旨，一心为民。人民是我们的衣食父母，人民政府必须服务人民，全体公务人员要始终牢记政府前面的“人民”二字，自觉对人民负责。要眼睛向下、深入基层，通过开展“三解三促”(31)、公开接访、帮困解难等途径，倾听民声、了解民意，扎扎实实为群众解决问题。

我们一定履职尽责，敢于担当。面对全市人民的新期待，全体公务人员要切实加强学习，不断提升职业水准，用创新的思路和办法解决难题、推动发展；要切实负起责任，敢抓敢管敢担当，说了算、定了干、干则成，坚决纠正“为官不为”和“慵懒散”现象；要切实锤炼作风，组织开展第三期政府公务人员军训活动，锤炼令行禁止、雷厉风行的作风。

我们一定转变职能，阳光高效。认真落实行政审批制度改革各项清单，推进行政权力100%上网运行。试点“三证合一”(32)，简化流程手续，提高服务效率。创建国家级行政服务标准化示范单位。对涉及审批的中介机构加强规范管理。强化政府信息公开和新闻发布工作。市级部门除涉密的以外，全部公开部门预决算和“三公”经费预决算。

我们一定依法行政，科学民主。自觉接受市人大及其常委会的法律监督、工作监督和市政协的民主监督，主动接受社会公众和新闻舆论监督。认真落实重大事项向市人大报告制度，充分发挥市政协和各民主党派、工商联、无党派人士以及人民团体的参政议政作用，切实提高人大建议和政协提案的办成率。推进各级领导干部学法常态化，建立健全法律顾问制度。重大行政决策认真执行公众参与、专家论证、风险评估、合法性审查和集体讨论等法定程序。

我们一定从严治政，清正廉洁。严格执行中央“八项规定”和省委、市委“十项规定”，巩固提升党的群众路线教育实践活动的成果。严控一般性支出，保持机关事业单位实有人员负增长，完成党政机关公务用车改革。认真落实党风廉政建设责任制，加大暗访督查、行政监察、审计监督力度，严厉查处各种违法违纪行为，树立为民、务实、清廉的良好形象。

各位代表，“镇江很有前途”的寄语鼓舞人心、催人奋进!让我们牢记习总书记的殷切期望，在省委、省政府和市委的正确领导下，把上级要求结合镇江实际贯彻好，把人民群众需要我们做的事情落实好，把确定下来的目标任务完成好，奋力开创生态领先特色发展的新局面，为建设经济强、百姓富、环境美、社会文明程度高的新镇江而努力奋斗!入市。加强农村集体“三资”管理，推进社区股份制改革；加快发展新型集体经济。
